# A Sustainable Approach for the Use of Plant Proteins as Functional Ingredients in Dairy Alternatives

**DOI:** 10.1002/fsn3.72403

**Published:** 2026-09-23

**Authors:** Boutheina Ben Akacha, Monika Michalak, Miroslava Kačániová, Maciej Ireneusz Kluz, Wirginia Kukula‐Koch, Wojciech Koch, Ramona Suharoschi, Rania Ben Saad, Stefania Garzoli, Anis Ben Hsouna

**Affiliations:** ^1^ Laboratory of Biotechnology and Plant Improvement, Centre of Biotechnology of Sfax Sfax Tunisia; ^2^ Department of Pharmaceutical Sciences, Collegium Medicum Jan Kochanowski University Kielce Poland; ^3^ Institute of Horticulture, Faculty of Horticulture and Landscape Engineering Slovak University of Agriculture Nitra Slovakia; ^4^ School of Medical & Health Sciences VIZJA University Warszawa Poland; ^5^ Andrzej Frycz Modrzewski Krakow University Kraków Poland; ^6^ Department of Pharmacognosy With Medicinal Plants Garden Medical University of Lublin Lublin Poland; ^7^ Department of Food and Nutrition Medical University of Lublin Lublin Poland; ^8^ University of Agricultural Sciences and Veterinary Medicine Cluj‐Napoca Cluj‐Napoca Cluj Romania; ^9^ Department of Chemistry and Technologies of Drug Sapienza University Rome Italy; ^10^ Department of Environmental Sciences and Nutrition, Higher Institute of Applied Sciences and Technology of Mahdia University of Monastir Monastir Tunisia

**Keywords:** dairy alternatives, extraction technologies, food innovation, nutritional quality, protein functionality, sustainable food systems

## Abstract

The increasing global demand for sustainable and health‐promoting foods has positioned plant‐based proteins as key components in the development of dairy alternatives. This review critically examines plant protein sources: legumes, cereals, pseudocereals, oilseeds, and nuts not merely by composition, but by their comparative suitability for specific dairy applications, evaluating protein quality using the Digestible Indispensable Amino Acid Score (DIAAS), digestibility, and source‐specific antinutritional factors (phytates in cereals; tannins, trypsin inhibitors, and lectins in legumes and oilseeds) relative to milk‐derived reference proteins. Structural determinants of functional performance particularly the relative proportion of 7S versus 11S globulin fractions are identified as a stronger predictor of emulsifying, foaming, and gelling behavior than protein source or purity alone, while extraction method emerges as a recurring confounding variable, since conventional alkaline and acid extraction routes measurably compromise the same structural integrity that governs functional performance. Conventional and innovative extraction technologies (ultrasound‐, pulsed‐electric‐field‐, deep‐eutectic‐solvent‐, and enzyme‐assisted extraction) are compared on this basis, alongside their trade‐offs between yield, structural preservation, and industrial scalability. Regulatory constraints on dairy‐product naming in the EU and beyond, and the competitive landscape of plant‐based dairy manufacturers including a shift toward precision‐fermentation and mycoprotein‐based approaches, are also examined. This review further highlights a methodological limitation affecting the underlying evidence base: the absence of standardized functional‐property assays across studies means that a meaningful share of reported “source‐driven” variability may instead reflect protocol‐driven inconsistency, a caveat that should inform how findings across this literature are compared. Collectively, this review provides an evidence‐based, application‐oriented framework for selecting and processing plant proteins to meet the specific nutritional, functional, and regulatory demands of dairy‐alternative formulation.

## Graphical abstract

Plant proteins in dairy alternatives.

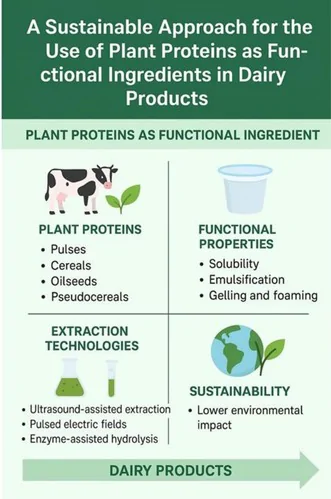

## 1 Introduction

As the world's population grows and environmental problems increase, the food industry is under increasing pressure to develop sustainable, nutritious, and ethical protein alternatives. Plant‐based proteins have proven to be valuable candidates in this transition process (Akacha et al. 2026; Sabaghi and Seyedalmoosavi 2025). Not only do they offer environmental advantages over animal‐based proteins, such as lower greenhouse gas emissions and less land and water use, but they also offer multiple nutritional and functional benefits (Bryant 2022). Plant proteins, derived from various sources such as legumes, grains, oilseeds, pseudocereals, and nuts, are rich in essential nutrients and bioactive compounds. However, their nutritional quality often depends on amino acid composition, digestibility, and the presence of antinutritional factors (Nandan et al. 2024).

Recently, plant proteins have become increasingly crucial in formulating plant‐based dairy alternatives such as milk, yogurt, and cheese. Their successful integration depends on their nutritional value and functional properties, such as solubility, emulsification, gelling, and foaming, which vary greatly depending on the protein source and processing method (Bryant 2022; Alehosseini et al. 2025). Both traditional and innovative protein extraction methods are used to optimize these properties. Conventional methods such as alkali or acid extraction are still widely used, but have limitations regarding yield, quality, and sustainability. Novel techniques such as ultrasound‐assisted extraction, pulsed electric fields, enzyme‐assisted processes, and deep eutectic solvents offer promising alternatives that meet the requirements of clean label and green processing (Nikbakht Nasrabadi et al. 2021).

While existing reviews have extensively cataloged plant protein sources, their nutritional composition, and general functional properties (Langyan et al. 2021; Kaur et al. 2023), this review's contribution lies in an application‐specific, comparative framework addressing a question largely unanswered in this literature: which plant protein, processed by which method, is suited to which dairy application, and why. This review advances the field in three specific ways. First, it identifies the relative proportions of 7S and 11S globulin fractions as a mechanistic explanation for divergent emulsifying, foaming, and gelling performance across sources, moving beyond descriptive reporting of functional variability toward an explanatory basis for it. Second, it explicitly identifies extraction method as a recurring confounding variable in the functional‐properties literature, a distinction with direct consequences for interpreting and comparing published data across studies. Third, it critically appraises a structural limitation in the underlying evidence base itself: the absence of standardized functional‐property assay protocols which constrains direct cross‐study comparisons until such protocols are more widely adopted. Building on established DIAAS‐based comparisons of plant and dairy protein quality (Herreman et al. 2020), this review integrates protein‐quality benchmarking with these structural and methodological findings into a single application‐specific decision framework, consolidating evidence that has previously existed only in separate strands of the literature. Taken together, these elements position this review as a decision‐oriented framework for dairy‐alternative formulation, rather than a further descriptive compilation of plant protein literature.

## 2 Methods: Literature Search Strategy

This narrative review synthesizes 92 peer‐reviewed sources published between 2003 and 2026, with the substantial majority (80%) published from 2021 onward, reflecting the recent acceleration of research on plant protein extraction and functionality for dairy‐alternative applications. Search terms combined “plant protein” OR “plant‐based protein” with “dairy alternative,” “dairy analogue,” “extraction,” “functional properties,” “emulsifying,” “foaming,” “gelling,” or “solubility,” restricted to English‐language publications, across Scopus, Web of Science, PubMed, and ScienceDirect. Sources were retained based on direct relevance to plant protein sources, extraction technologies, functional properties, or applications in dairy alternatives; studies addressing plant proteins exclusively in non‐dairy contexts (e.g., meat analogs, bakery products) were excluded unless the underlying functional data were directly transferable to dairy‐analog formulation. Regulatory and market information was supplemented with company websites, EUR‐Lex, and industry sources, accessed in May 2026.

## 3 The Rise of Plant‐Based Proteins in Dairy Alternatives

**FIGURE 1:**
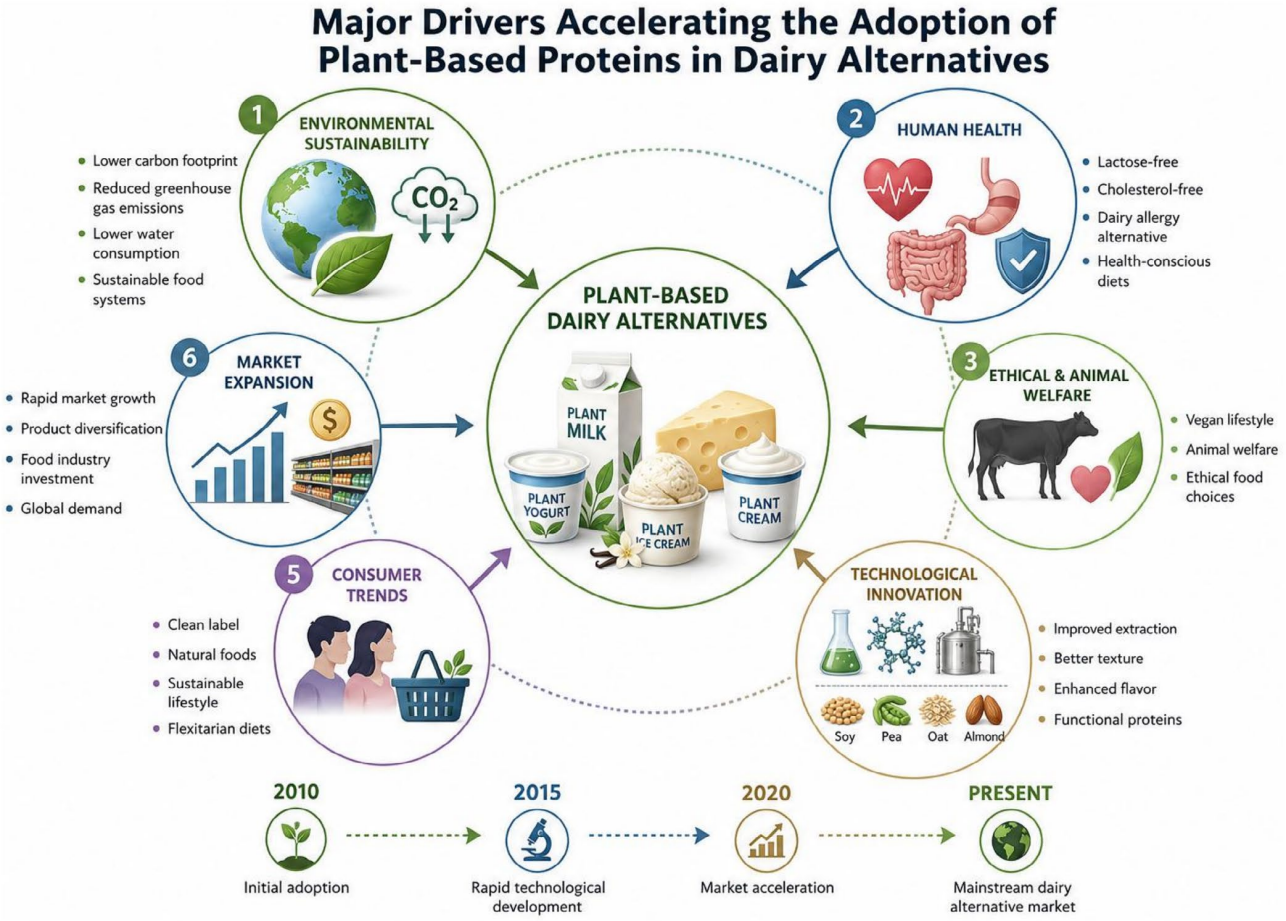
Conceptual framework of the convergent driver's environmental sustainability, human health, ethical and animal welfare, technological innovation, consumer trends, and market expansion accelerating the adoption of plant‐based proteins in dairy alternatives, and their translation into market growth from 2010 to present.

The application of plant protein to dairy gained greater popularity at the beginning of the 2010s, and the momentum accelerated from 2015 onwards (Kumar et al. 2022). This is the effect of various factors. Awareness of environmental problems has played a large role, with dairy production associated with a high carbon impact, using excess water, and producing greenhouse gasses. Plant‐based alternatives are generally associated with a lower per‐unit‐protein environmental footprint, though the magnitude of this difference varies by crop, cultivation region, and processing method, providing a broader synthesis of these comparative findings across multiple plant protein sources (Warren et al. 2023). Health concerns have also contributed to this shift, as studies have uncovered potential adverse effects of consuming dairy, such as lactose intolerance, allergies, and concerns related to elevated blood cholesterol levels and heart disease, which have led consumers to seek healthier alternatives (Silva et al. 2020). In addition, the growing trend toward veganism and flexitarians has encouraged consumers to reduce consumption of animal products for ethical, environmental, or animal welfare reasons (Sheen et al. 2023). Improved plant protein processing technology, including soy, oats, almonds, and peas, has produced alternatives with tastes and feels like traditional dairy. In addition, the shifting lifestyle of consumers, particularly among the Millennial and Generation Z segments, opts for natural, ethical, and sustainable food. Since 2020, the plant‐based dairy industry—covering plant‐based milk, yogurt, cheese, and cream—has continued a growth path, driven by increasing consumer demand and investment from the food industry (Warren et al. 2023). Figure 1 provides a conceptual overview of the key factors driving the rapid adoption of plant‐based proteins in dairy alternatives. It integrates environmental, health, ethical, technological, consumer, and market‐related drivers while illustrating the progressive evolution of the sector from initial adoption to mainstream commercialization.

More recent industry data indicate that this growth trajectory has become more complex since 2024. Market analysts now describe the sector as entering a “reset and rebalancing phase” following its rapid expansion in the early 2020s, as the category has matured and confronted pricing inflation, ultra‐processing concerns, and persistent taste and functionality gaps relative to conventional dairy (Giantsidi and Tarantola 2025). Market size estimates for 2025–2026 vary considerably by research methodology, ranging from approximately $25–27 billion (Future Market Insights, 2026; Transparency Market Research, 2026) to $37–41 billion (Madsen et al. 2025), with most sources converging on a compound annual growth rate of roughly 7%–13% through the early 2030s. Growth is notably uneven across product categories: U.S. retail tracking recorded a 10% decline in plant‐based cheese dollar sales and an 8% decline in unit sales in 2025, reflecting the mouthfeel and melt performance limitations discussed in Section 10, whereas plant‐based milk has continued to grow, with almond milk holding the largest unit sales share (49%) ahead of oat (21%) and soy (8%) in the United States. Adoption of Western‐style plant‐based foods (Short et al. 2021; Warner et al. 2025). This shift toward almond dominance in the beverage category, alongside soy's continued lead in the broader dairy‐alternatives market by value, illustrates the divergence between commodity‐scale ingredient use and premium, nutrition‐forward positioning. Several major brands have responded by launching protein‐fortified products; for example, Califia Farms introduced a protein‐fortified almond milk delivering 8 g of protein per serving in 2025, explicitly targeting consumers seeking nutritional parity with dairy (de Andrade et al. 2026). Geographically, Asia Pacific now leads the plant‐based milk market by revenue share (47.6%), with China identified as the fastest‐growing regional market (16.2% CAGR), attributed to established cultural familiarity with soy and tofu products combined with rising urban adoption of Western‐style plant‐based foods (Asif et al. 2026).

## 4 Natural Sources and Classification of Plant‐Based Proteins

### 4.1 Classification Based on the Solubility and Structural Properties of Plant‐Based Proteins

Plant‐based proteins are derived from various natural sources, each offering distinct structural and functional properties. Primary sources include legumes (such as beans, lentils, peas, chickpeas, and soybeans), oilseeds (like sunflower, rapeseed, flaxseed, and hemp seed), nuts (including almonds, cashews, walnuts, and peanuts), whole grains (such as quinoa, oats, rice, and wheat), and tubers like potatoes. These plant proteins are classified based on their solubility and biochemical characteristics, following Osborne's classical system, which divides them into four major fractions: albumins, globulins, prolamins, and glutelins (Craig et al. 2023).

Albumins are water‐soluble proteins primarily found in legumes, oilseeds, and pulses rather than cereals. Upon ultracentrifugation, albumins can be separated into three fractions: 2S, 7S, and 11S. The 2S fraction is the most prevalent and primary storage protein in many plant species, particularly legumes, lupins, and peas. These proteins are characterized by a high content of sulfur‐containing amino acids, including methionine and cysteine, which play a crucial role in protein stability and functionality (Yang et al. 2024).

Globulins, which are soluble in salt solutions but insoluble in water, are abundant in legumes and present in both dicot and monocot plants, as well as in some gymnosperms. Compared to albumins, globulins are deficient in sulfur‐containing amino acids. They are further classified into 7S and 11S fractions, which differ in molecular weight and functional properties. Examples of globulin proteins include β‐conglycinin (7S) and glycinin (11S) in soybean, β‐conglutin (7S) and α‐conglutin (11S) in lupins, legumin (11S), vicilin (7S), and convicilin (7S) in peas, helianthinin (11S) in sunflower seeds, and cucurbitin (11S) in Cucurbita species (Kimura et al. 2008; Mistry et al. 2022).

Prolamins are a class of plant proteins predominantly found in cereals, except for oats and rice (Sha et al. 2024). These proteins are soluble in aqueous ethanol solutions but insoluble in pure water or alcohol. Historically, they were first identified as gliadins, but Osborne proposed the term “prolamins” due to their high proline and glutamine content. Prolamins generally have low levels of arginine and histidine, with lysine often absent. They are classified into different categories based on their amino acid composition and molecular structure. For instance, prolamins in wheat and rye are known as gliadins, in barley as hordeins, in sorghum as kafirins, and in maize as zeins, which include α, β, γ, and δ fractions (Day 2013). Prolamins in oats are known as avenins. Within Triticeae cereals (wheat, barley, rye), prolamins are further divided into sulfur‐rich prolamins, which contain high levels of proline, glutamine, and cysteine with disulfide bonds; high‐molecular‐weight (HMW) prolamins, which are rich in glycine and glutamine; and sulfur‐poor prolamins, which include class 1 to 3 prolamins as major fractions and class 4 prolamins as minor fractions, though rich in sulfur‐containing amino acids like methionine and cysteine (Hassan et al. 2022).

Glutelins, another major class of plant proteins, are found in various grass species and are particularly abundant in cereals. Unlike other protein fractions, glutelins are insoluble in water, salt solutions, or alcohol but can be dissolved in dilute acid or alkaline solutions (Zhang, Boateng, et al. 2024). Gluten is a notable example of glutelins, a mixture of prolamins and glutelins found in wheat. Within gluten, gliadins represent the prolamin fraction, while glutenins constitute the glutelin fraction. Gliadins are monomeric proteins, while glutenins are polymeric and form extensive networks through intrachain disulfide bonds. Glutenins are further divided into high‐molecular‐weight (HMW) and low‐molecular‐weight (LMW) subunits, which contribute to the viscoelastic properties of wheat dough, making them essential in baking applications (Day 2013).

This classification system provides a comprehensive understanding of plant‐based proteins, enabling their targeted application in food formulations (Li et al. 2025). The solubility and structural differences among albumins, globulins, prolamins, and glutelins influence their digestibility, nutritional profile, and functional properties in plant‐based food products. Advances in protein extraction and modification techniques continue to enhance the application of these plant proteins in food industries, particularly in developing sustainable and nutritious alternatives to animal‐derived proteins (Oluwajuyitan and Aluko 2025).

### 4.2 Classification Based on the Sources of Plant‐Based Proteins

**FIGURE 2:**
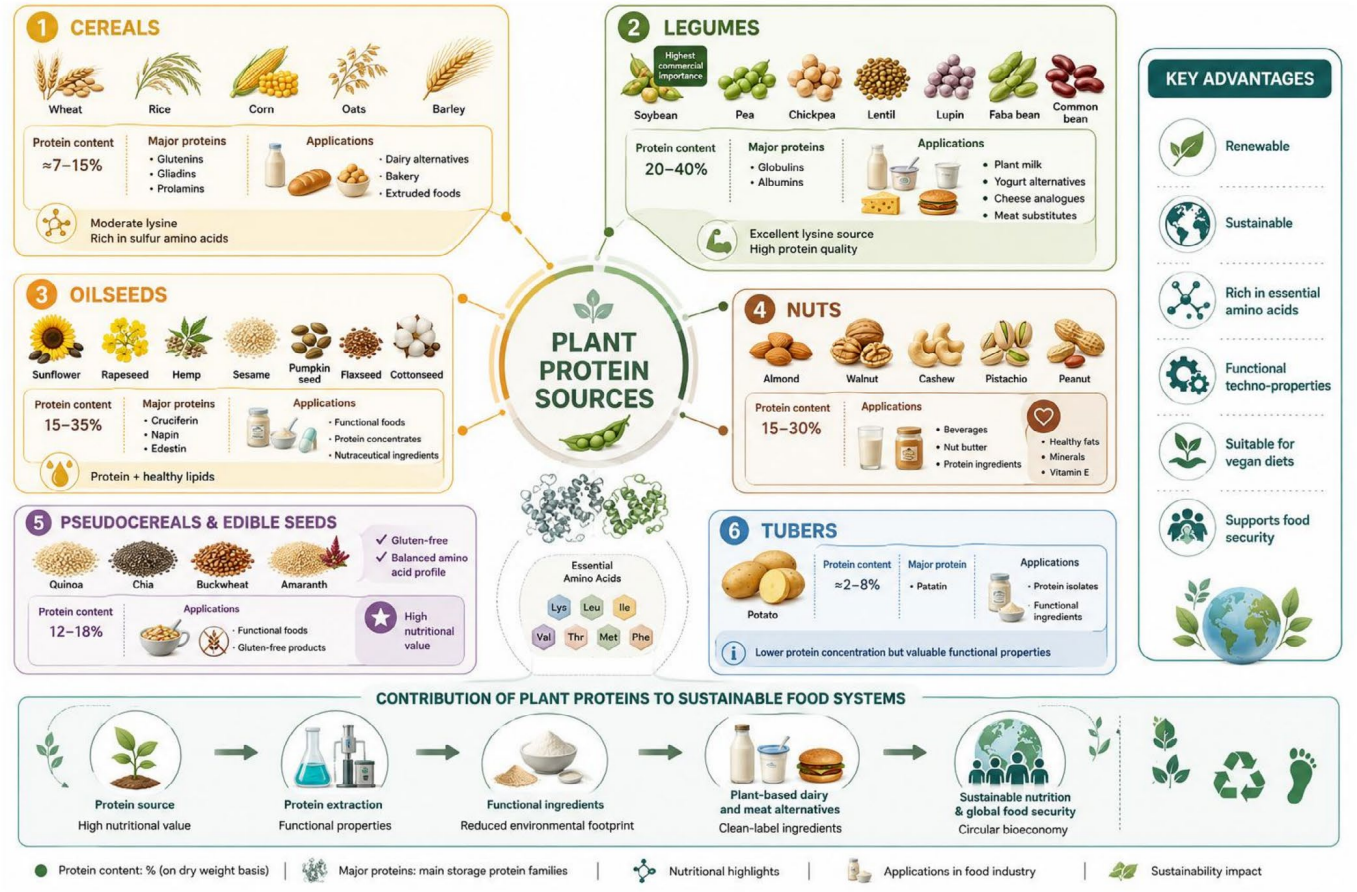
Classification, nutritional characteristics, and food applications of major plant protein sources.

Proteins of plant origin represent a diverse and nutritionally valuable source of essential amino acids, making them an essential component of sustainable diets. These proteins can be classified into distinct groups according to their botanical origin and composition (Langyan et al. 2021) (Figure 2). Cereals, including wheat, corn, oats, rice, and barley, contain moderate protein levels, with wheat being particularly rich in gluten. Legumes, such as soybeans, peas, beans, chickpeas, lentils, lupins, and faba beans, are among the most protein‐dense plant sources widely used in dairy products and plant‐based meat substitutes. Oilseeds, including sunflower, rapeseed, linseed, hemp seed, cottonseed, sesame seed, and pumpkin seed, provide protein and beneficial lipids, contributing to their application in functional foods. Nuts, such as almonds, pistachios, cashews, walnuts, and peanuts, offer a combination of protein, healthy fats, and essential micronutrients (Luigia 2019).

Edible seeds and pseudocereals, including quinoa, buckwheat, chia seed, and amaranth, are gluten‐free protein sources with a well‐balanced amino acid profile, making them suitable for specialized diets. Although tubers like potatoes are not primarily protein‐rich, they still contribute to plant‐based formulations due to their starch‐protein matrix. Given their diverse nutritional profiles and functional properties, plant‐based proteins are increasingly recognized for their potential innovation, particularly in developing sustainable protein alternatives to support global food security (Balakrishnan and Schneider 2022).

Table 1 classifies plant‐based proteins into distinct categories by botanical origin and protein composition. This classification framework helps understand plant‐based proteins' nutritional and functional diversity and their role in food applications.

**TABLE 1:** Summary of plant‐based protein sources: Botanical origin, protein content, and applications.

Category	Examples	Scientific name	Protein content (%)	Dairy‐alternative application	References
Cereals	Wheat, Corn, Oat, Rice, Barley	*Triticum* spp., *Zea mays*, etc.	8%–15%	Oat‐ and rice‐based milk beverages (e.g., Oatly); starch fraction contributes texture and viscosity in beverage‐format dairy alternatives	Danciu et al. (2023)
Legumes	Soy, Peas, Beans, Chickpeas, Lentils, Lupins, Faba beans	*Glycine max, Pisum sativum*, etc.	20%–40%	Primary protein backbone for structured dairy alternatives: soy‐ and pea‐based milk, yogurt, and cheese analogs	Zhang, Boateng, et al. (2024), Zhang, Zhang, et al. (2024)
Oil Seeds	Sunflower, Rapeseed, Flaxseed, Hemp seed, Cotton seed, Sesame seed, Pumpkin seed	*Helianthus annuus, Brassica napus*, etc.	18%–25%	Hemp‐ and flaxseed‐based milk beverages (e.g., Good Karma Foods); lipid fraction contributes to fat‐matrix formulation in plant‐based creamers and cheese.	González‐Pérez and Arellano (2009)
Nuts	Almond, Pistachio, Cashew, Walnut, Peanut	*Prunus dulcis, Arachis hypogaea*, etc.	15%–30%	Dominant nut‐derived category: almond‐based milk beverages (highest‐volume segment) and cashew‐based cheese analogs (e.g., Miyoko's Creamery, New Roots).	Aydar et al. (2020)
Edible seeds and pseudocereals	Quinoa, Buckwheat, Chia seed, Amaranth	*Chenopodium quinoa, Fagopyrum esculentum*, etc.	12%–20%	Minor/emerging role as texturizing or nutritional‐fortification agents in dairy‐alternative beverages and puddings; not yet a primary protein source in commercial products.	Atudorei et al. (2026)
Tubers	Potato, Sweet Potato, Cassava, Taro, Yam	*Solanum tuberosum, Ipomoea batatas*, etc.	1%–6%	Potato protein used as a fortification ingredient in oat‐based milk alternatives; starch fraction contributes to texture in dairy‐analog matrices	Shewry (2003), McCarron et al. (2024)
Processed/Derived forms	Tofu, Tempeh, Seitan, Protein Isolates	Various	30%–80%	Protein isolates form the functional backbone of most commercial dairy‐analog formulations; tofu‐derived matrices used in cheese‐analog applications.	Auñon‐Lopez et al. (2025)

### 4.3 Other Classifications of Plant‐Based Proteins

Plant‐based proteins can be classified based on various criteria, highlighting different characteristics relevant to their functionality and applications.

From a nutritional standpoint, proteins can be classified as complete (containing all essential amino acids, such as quinoa and soy) or incomplete (lacking one or more essential amino acids, such as wheat or lentils), with some being particularly rich in lysine (e.g., legumes) or sulfur‐containing amino acids (e.g., cereals) (Kumar et al. 2022).

The classification based on functionality groups proteins according to their technological properties: gelling proteins, such as pea protein isolate, which forms heat‐induced gels in a pH‐ and ionic‐strength‐dependent manner (Zhang, Boateng, et al. 2024); emulsifying proteins, such as the 7S globulin fractions of pea, fava bean, cowpea, and French bean, which display comparatively strong interfacial properties relative to soybean 7S globulin (Kimura et al. 2008); foaming proteins, such as faba bean/hemp protein blends, whose foaming capacity varies systematically with blend ratio and rice bran protein nanoparticles, whose foam stability depends on interfacial molecular structure (Zhang, Boateng, et al. 2024); and water‐binding proteins, such as oat protein.

Furthermore, proteins can be classified based on their treatment and modification, distinguishing between native proteins (untreated, such as raw legumes), hydrolyzed proteins (partially decomposed for better digestibility), isolates (highly purified extracts like soy protein isolate), concentrates (less refined with more fiber), and fermented proteins (such as tempeh and miso, known for their improved bioavailability) (Náthia‐Neves et al. 2025).

Industrial applications further define plant proteins as those used in food production (e.g., plant‐based meat, dairy alternatives), pharmaceutical and nutraceutical products (protein supplements, medical nutrition), cosmetics (oat and rice proteins for skin and hair care), and animal feed (sunflower or rapeseed meal) (Hu et al. 2022).

### 4.4 Selection Criteria and Comparative Suitability of Plant Proteins for Dairy Applications

While the classifications above describe the structural and compositional diversity of plant proteins, translating this diversity into functional dairy alternatives requires an explicit, comparative assessment of source‐dependent advantages and limitations. Soy protein remains the most extensively used source for dairy analogs owing to its high solubility, strong gelling and emulsifying capacity, and cost‐effective large‐scale isolation; however, its residual beany flavor and status as a major allergen restrict its use in hypoallergenic or flavor‐sensitive product lines. Pea protein has emerged as a leading soy alternative for dairy‐free yogurts and beverages because of its non‐allergenic profile and favorable amino acid balance, yet its comparatively poor solubility at neutral pH and pronounced off‐notes (attributed to lipoxygenase‐derived volatiles) necessitate additional debittering or masking steps before incorporation into consumer‐facing products (Anyiam et al. 2025). Oat protein offers a naturally gluten‐adjacent, mild‐flavored matrix well suited to fermented dairy alternatives such as oat‐based yogurt, but its lower protein density and weaker gel network compared to legume proteins often require blending with hydrocolloids or other proteins to achieve acceptable texture. Rice protein, valued for its hypoallergenicity and neutral taste, is frequently used in combination with pea or soy protein to offset its inherently poor solubility and limited lysine content (Zhang, Boateng, et al. 2024). Almond and other tree‐nut proteins are increasingly applied in premium dairy‐alternative beverages for their favorable lipid co‐extraction and sensory profile, though their comparatively low protein yield and tree‐nut allergen status limit broader applicability. Selecting an appropriate plant protein for a given dairy application therefore depends on matching source‐specific functional attributes, solubility across the relevant pH range, gelation, heat stability under pasteurization conditions, flavor neutrality, allergenicity, and extraction scalability to the technical requirements of the target matrix (fluid milk analog, spoonable yogurt, or semi‐hard cheese analog), rather than on protein content alone (Asif et al. 2026).

## 5 Biochemical Composition and Nutritional Value of Plant‐Based Proteins

Plant‐based protein sources vary widely not only in macronutrient composition but, more critically, in protein quality, a distinction that broad compositional ranges alone do not capture. Protein quality is best assessed using the Digestible Indispensable Amino Acid Score (DIAAS), the metric currently recommended by the FAO over the older Protein Digestibility‐Corrected Amino Acid Score (PDCAAS), as DIAAS accounts for the true ileal digestibility of individual amino acids rather than assuming uniform digestibility across the whole protein (Hertzler et al. 2020). Table 2 presents DIAAS values for representative plant protein sources relevant to dairy‐alternative formulation, benchmarked directly against skim milk powder as the dairy‐protein reference.

**TABLE 2:** Digestible Indispensable Amino Acid Scores (DIAAS) of representative plant protein sources relevant to dairy alternatives, benchmarked against a dairy protein reference (adult values shown).

Source	DIAAS – Adults (%)	Limiting amino acid	FAO quality classification	References
Skim milk powder (dairy benchmark)	131	—	Excellent (> 100)	Oh and Kim (2025)
Soy protein isolate	87	Sulfur amino acids (Met + Cys)	Good (75–100)	Oh and Kim (2025)
Chickpea (cooked)	76	Sulfur amino acids	Good (75–100)	Han et al. (2020)
Pea protein concentrate	69	Sulfur amino acids	No claim (< 75)	Oh and Kim (2025)
Oat protein concentrate	67	Lysine	No claim (< 75)	Brückner‐Gühmann et al. (2019)
Wheat protein	66	Lysine	No claim (< 75)	Oh and Kim (2025)
Fava bean (cooked)	60	Lysine	No claim (< 75)	Han et al. (2020)
White rice protein	60	Lysine	No claim (< 75)	Oh and Kim (2025)

Using the FAO‐recommended DIAAS classification, a value above 100 qualifies a protein as an excellent source, 75–100 as a good source, and below 75 as insufficient to support an independent protein‐quality claim. Among the sources evaluated, only skim milk powder exceeds the “excellent” threshold, while soy protein isolate is the sole plant‐derived protein to reach “good” status; pea, oat, wheat, and rice‐derived proteins all fall below the 75‐point threshold, most commonly limited by lysine or sulfur amino acid content (Brückner‐Gühmann et al. 2019). This has a direct practical implication for dairy‐alternative formulation: rather than relying on a single plant protein source, blending complementary proteins for example, pairing a lysine‐limited cereal protein (wheat, rice, oat) with a legume protein relatively richer in lysine, such as soy or pea is generally required to approach the amino acid completeness of the milk protein it is intended to replace. This quality‐based comparison provides a more actionable basis for ingredient selection than the broad compositional ranges alone, directly informing the source‐selection criteria discussed in Section 4.4.

Table 3 (below) provides supplementary reference data on the general macronutrient composition of whole grains, pseudo‐cereals, legumes, oilseeds, and nuts; the comparative protein‐quality assessment relevant to dairy‐alternative formulation is presented in Table 2 above.

**TABLE 3:** Nutritional composition of whole grains, pseudo‐cereals, legumes, oilseeds, and nuts (per 100 g).

Category	Food item	Energy (kcal)	Protein (g)	Fat (g)	Carbohydrates (g)	Fiber (g)	Notable nutrients (mg)	References
Cereals	Oats	389	16.9	6.9	66.3	10.6	Iron (4.7), Magnesium (177)	Garutti et al. (2022)
Cereals	Brown Rice	362	7.5	2.7	76.2	3.5	Selenium (15), Manganese (1.8)	Garutti et al. (2022)
Legumes	Soybeans	446	36.5	19.9	30.2	9.3	Isoflavones, Calcium (277)	Shevkani (2023)
Legumes	Pea	341	24.6	1.2	60.4	25.5	Folate (0.274), Iron (4.4)	Morris and Mohiuddin (2026)
Legumes	Chickpeas	364	19.3	6.0	60.7	17.0	Potassium (875), Zinc (3.4)	Shevkani (2023)
Oilseeds	Flaxseeds	534	18.3	42.2	28.9	27.3	Omega‐3 (22.8 g), Lignans	Jardim et al. (2024)
Oilseeds	Sunflower Seeds	584	20.8	51.5	20.0	8.6	Vitamin E (35.2), Selenium (53)	Jardim et al. (2024)
Nuts	Almonds	579	21.3	50.6	21.6	12.5	Vitamin E (25.6), Magnesium (268)	Jardim et al. (2024)
Nuts	Cashews	553	18.2	46.4	30.2	3.3	Zinc (5.8), Iron (6.7)	Jardim et al. (2024)

Beyond compositional differences, essential amino acid (EAA) content is a key determinant of whether a plant protein source can support dairy‐equivalent nutritional claims, since the nine indispensable amino acids (histidine, isoleucine, leucine, lysine, methionine, phenylalanine, threonine, tryptophan, valine) cannot be synthesized by the human body and must be supplied by diet (Wilson and Bendich 2022; Auñon‐Lopez et al. 2025). Dairy proteins set a high benchmark: milk, whey, and casein contain approximately 40%, 35%, and 60% essential amino acids, respectively. Among the plant protein sources most relevant to dairy‐alternative formulation (Table 2), essential amino acid content reaches 27% for soy, 28% for rice, 30% for pea, and 32% for corn protein isolates meeting WHO/FAO/UNU recommended proportions individually, but still falling well short of the dairy benchmark (Kaur et al. 2023). On average, animal proteins exceed plant protein sources by approximately 39% in essential amino acid content (Guo et al. 2024). As established in Section 4, soy and pea are consequently the two plant sources most commonly blended in fortified plant‐based dairy products to approximate dairy‐equivalent amino acid completeness, whereas single‐source formulations using lower‐EAA proteins are more constrained in their ability to support nutritional‐parity claims without amino acid fortification.

## 6 Comparative Digestion and Absorption of Animal‐Based and Plant‐Based Proteins in the Human Body

Protein digestion proceeds through sequential oral, gastric, pancreatic, and small‐intestinal absorptive phases common to both animal‐ and plant‐derived proteins: mechanical breakdown by chewing, acid‐mediated denaturation and pepsin cleavage in the stomach, further hydrolysis by pancreatic proteases (trypsin, chymotrypsin, carboxypeptidase, elastase) in the duodenum, and final absorption of free amino acids and small peptides across the intestinal brush border (Patricia and Dhamoon 2026). While this sequence is structurally identical for both protein sources, the efficiency of digestion at each stage diverges substantially between plant and animal proteins, and among plant sources themselves for the reasons discussed below.

Digestibility differences between plant and animal proteins are not primarily a matter of amino acid chemistry but of physical and chemical accessibility. Animal proteins reach the small intestine largely unobstructed, whereas plant proteins are embedded within fibrous cell wall matrices cellulose, hemicellulose, and pectin that must first be disrupted before proteases can act, which is why overall plant protein digestibility (50%–90%) remains consistently lower and more variable than that of animal proteins (~90%–99%) (Guo et al. 2024).

This variability is driven substantially by antinutritional factors, whose type and concentration differ systematically by botanical origin. Cereals are characterized primarily by phytates and polyphenols, which chelate divalent minerals such as zinc, iron, and calcium and indirectly impair enzyme‐cofactor availability, whereas pulses and oilseeds carry a more digestibility‐relevant burden of tannins, trypsin inhibitors, and lectins that act directly on proteolytic enzymes and on dietary protein itself (Heda et al. 2025). These differences are not merely qualitative: germination of chickpeas, peas, mung beans, and lentils has been shown to reduce phytic acid content by up to 75.65% and trypsin inhibitor activity by up to 39.20%, demonstrating that antinutritional‐factor reduction is both source‐dependent and quantifiable rather than a uniform improvement across plant proteins (Nandan et al. 2024; Patricia and Dhamoon 2026). This source‐specific pattern also explains why industrially isolated proteins typically outperform raw or minimally processed forms in digestibility: soy protein isolate, for instance, retains higher digestibility (85%–90%) than raw legume flour, largely because isolation removes much of the fiber‐ and antinutritional‐factor‐rich matrix that limits digestibility in less‐refined forms.

Processing technologies substantially narrow this digestibility gap, though their effectiveness depends on matching the treatment to the dominant antinutritional factor present. Soaking and germination activate endogenous phytases and proteases that degrade phytate and denature protease inhibitors, making them particularly effective for cereal‐ and legume‐based sources where phytate or trypsin inhibitors dominate. Fermentation (as in tempeh or miso) achieves broader gains by using microbial enzymes to partially hydrolyze proteins and degrade a wider range of antinutritional compounds simultaneously. Thermal processing (extrusion, heat treatment) is most effective against heat‐labile factors such as trypsin inhibitors and lectins, though excessive heat can conversely reduce digestibility through Maillard‐type cross‐linking that lowers lysine bioavailability (Kiela and Ghishan 2016). Selecting a processing strategy therefore requires matching the treatment to the specific antinutritional profile of the source protein; a cereal‐dominant phytate burden calls for phytase‐activating treatments, while a legume‐dominant trypsin‐inhibitor burden benefits more directly from heat treatment rather than applying a single generic processing method across all plant protein types.

During the absorption phase, peptidases at the brush border membrane of the small intestine hydrolyse the remaining dipeptides and tripeptides to free amino acids, which are then transported into the enterocytes via sodium‐dependent and proton‐coupled amino acid transporters. These amino acids enter the hepatic portal circulation and are transported to the liver, where they undergo metabolic processing, including protein synthesis, gluconeogenesis, and energy production. Animal‐based proteins are considered nutritionally superior due to their complete amino acid profile. In contrast, most plant‐based proteins are deficient in one or more essential amino acids, so supplementation or blending with complementary protein sources is required to ensure adequate intake (Kiela and Ghishan 2016).

**FIGURE 3:**
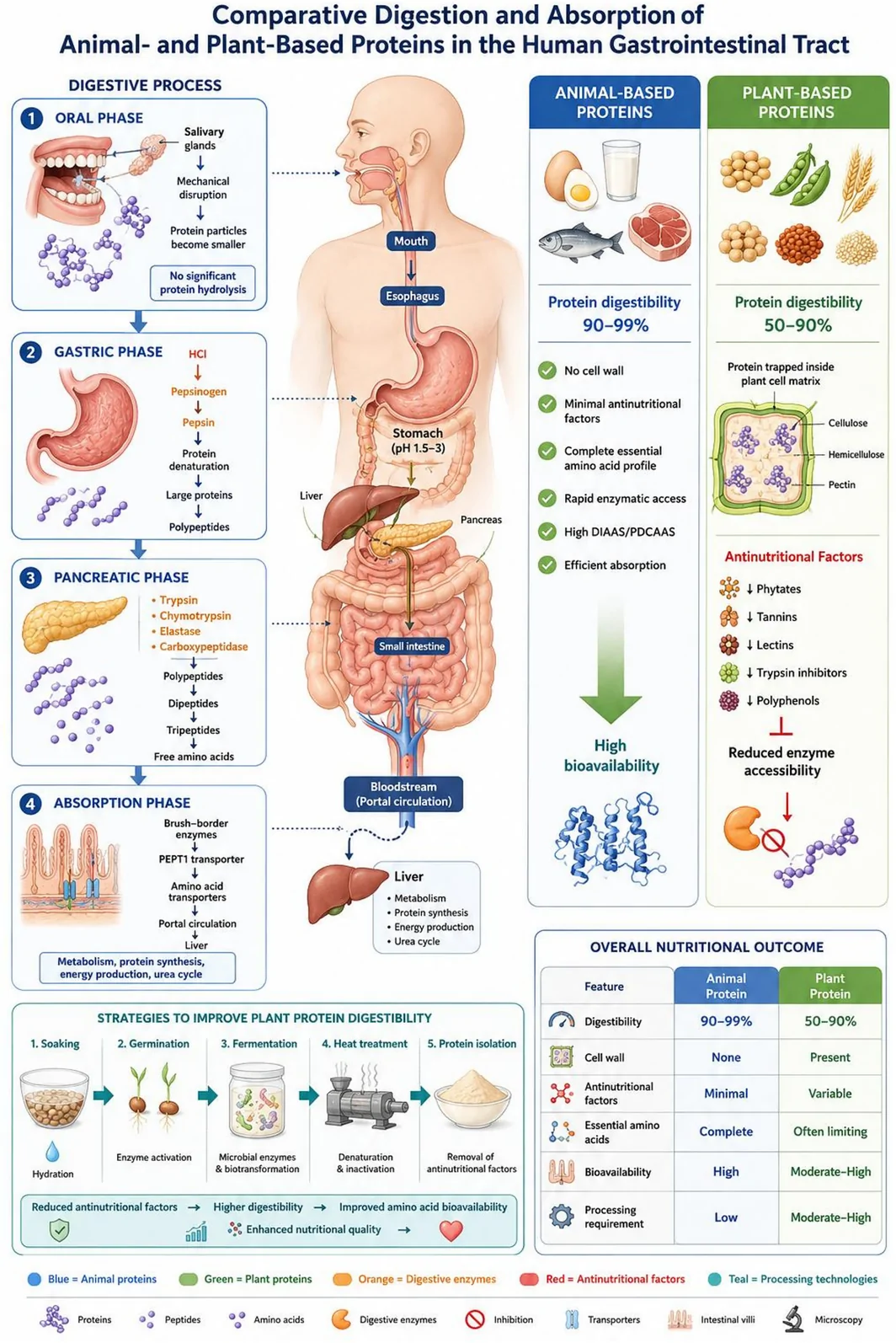
Mechanisms of gastrointestinal digestion and absorption of animal and plant proteins: Implications for protein bioavailability.

Excess amino acids, regardless of their source, cannot be stored. They are deaminated in the liver, producing ammonia, which is converted to urea via the urea cycle and excreted via the kidneys. However, plant protein consumption is associated with lower nitrogen storage, which may influence muscle protein synthesis and metabolic efficiency (Holeček 2022). Despite these differences (Figure 3), both animal and plant proteins contribute to human nutrition. Processing techniques such as fermentation, soaking, sprouting, and heat treatment can improve the bioavailability of plant proteins by reducing anti‐nutritional factors and improving digestibility. A balanced nutritional approach that incorporates different protein sources is essential to meet physiological protein requirements while considering sustainability and health aspects (Sharma, Zhang, and Rawdkuen 2025).

## 7 Key Players in the Global Plant‐Based Dairy Alternatives Market

Plant‐based dairy alternatives have gained significant traction globally, driven by consumer demand for sustainable, allergen‐free, and clean‐label options (Table 4). Beyond simple market presence, the competitive landscape reveals distinct technological and formulation strategies that are closely tied to the functional properties of the protein source used. Nut‐ and legume‐based systems exemplified by Miyoko's Creamery (USA) and New Roots (Switzerland) for cashew‐based cheeses, and Silverback Protein (Netherlands) for fava bean derivatives rely primarily on the gelling and emulsifying capacity of globulin‐rich protein fractions, which allow the mimicry of the textural and meltability properties of dairy cheese through fermentation and controlled coagulation. Coconut‐ and oat‐based systems, represented by Rebel Kitchen (UK), Califia Farms (USA), and Oatly (Sweden), depend less on protein functionality and more on polysaccharide‐ and lipid‐based stabilization, which explains their prevalence in beverage and creamer formats rather than structured cheese analogs.

**TABLE 4:** Leading companies and their main products in the plant‐based dairy industry.

Company	Main products	Country
Miyoko's Creamery	Plant‐based cheeses and butters	USA
Rebel Kitchen	Coconut‐based milk, yogurt, and beverages	UK
Upfield	Plant‐based spreads, margarines, and cheeses	Netherlands
New Roots	Cashew‐based cheeses	Switzerland
Grashka	Plant‐based dairy alternatives	Germany
VeggWhite	Plant‐based egg whites	USA
Silverback Protein	Fava bean‐based protein products	Netherlands
Promyc	Fermented mycoprotein‐based products	Belgium
VEGITASTIC	Legume‐based plant‐based products	Austria
VGIL'S	Nut‐based cheeses	France
Free From That	Plant‐based egg substitutes	Spain
Cathedral City	Plant‐based mature cheddar cheese	UK
Kinda Co	Cashew nut‐based cheeses	UK
C'é Dairy?	Cashew‐based fermented cheeses	UK
Bute Island Sheese	Various plant‐based cheese alternatives	UK
Nurishh	Plant‐based cheese slices	France
Applewood	Vegan smoked cheese	UK
Violife	Plant‐based cheese alternatives	Greece
Califia Farms	Almond, oat, and coconut milk, creamers, and yogurts	USA
Good Karma Foods	Flaxseed‐based milk, yogurt, and creamers	USA
MALK Organics	Cold‐pressed almond, oat, and cashew milk	USA
Delamere Dairy	Plant‐based milk alternatives (Planted)	UK
Amy's Kitchen Inc.	Plant‐based meals, including dairy alternatives	USA
Atlantic Natural Foods LLC	Plant‐based proteins and seafood alternatives	USA
Perfect Day Inc.	Animal‐free dairy proteins used in various products	USA
Blue Diamond Growers	Almond‐based milk and related products	USA
Eden Foods	Soy and rice milk products	USA
Earth's Own	Oat and almond‐based milk products	Canada
Oatly	Oat‐based milk, yogurt, ice cream	Sweden
Alpro	Soy, almond, oat, and coconut milk, yogurt, cream	Belgium
Silk	Almond, soy, oat, and cashew milk, yogurt, creamers	USA
Ripple	Pea‐based milk, half & half, protein shakes	USA
NotCo	AI‐developed plant‐based milk (NotMilk)	Chile

A more significant technological shift is evident in companies moving away from conventional plant extraction altogether. Perfect Day Inc. (USA) exemplifies this transition, using precision fermentation with genetically engineered microorganisms to produce dairy‐identical whey and casein proteins without any plant or animal input, thereby decoupling protein functionality from botanical origin and enabling closer replication of true dairy behavior (e.g., foaming, meltability) than plant‐protein blends can typically achieve. Similarly, Promyc (Belgium) and comparable ventures are exploiting fermented mycoproteins, which offer a fibrous, high‐protein matrix distinct from both legume and cereal proteins and may present fewer of the off‐flavor and anti‐nutritional challenges associated with pulse‐derived ingredients. These developments indicate a broader industry trajectory: first‐generation plant‐based dairy products (nut/oat/soy milks and simple cheese analogs) are increasingly being supplemented by second‐generation approaches, such as precision fermentation and mycoprotein, that prioritize functional equivalence to dairy proteins over reliance on any single plant source. This shift carries direct implications for formulation science, as it reframes the central challenge from “which plant protein most closely approximates casein or whey” toward engineering functionality at the molecular level, regardless of biological origin. Collectively, these companies illustrate that the growth of the plant‐based dairy sector is being shaped not only by consumer demand but by the increasing convergence of extraction technology, protein source selection, and biotechnological innovation as complementary levers for closing the sensory and functional gap with conventional dairy.

## 8 Regulatory Framework on Dairy Product Naming and the Use of Plant Proteins in the EU and Beyond

European legislation strictly regulates the *naming* of dairy products rather than the addition of plant‐derived ingredients per se. Following the ruling of the Court of Justice of the European Union (CJEU) of 14 June 2017 (Case C‐422/16, *TofuTown*), Article 78 and Annex VII, Part III of Regulation (EU) No 1308/2013 reserve terms such as “milk,” “cheese,” “yogurt,” “butter,” and “cream” exclusively for products derived from mammary secretions, with only a closed list of long‐established exceptions (e.g., “coconut milk,” “ice cream”) permitted under Commission Decision 2010/791/EU (Leialohilani and de Boer 2020). Consequently, plant‐based products must be marketed under alternative terms such as “plant‐based beverage” or “plant‐based preparation” if they are to contain no dairy component. This restriction concerns product denomination and labeling rather than composition: EU law does not generally prohibit incorporating plant proteins into a product still marketed under a protected dairy name, provided it meets the compositional standard of identity for that name and any non‐dairy addition is clearly declared under Regulation (EU) No 1169/2011 (Lähteenmäki‐Uutela et al. 2021). A parallel, and distinct, regulatory controversy has unfolded in France around the naming of *meat and fish* analogs rather than dairy: decrees n° 2022‐947 (29 June 2022) and n° 2024‐144 (26 February 2024) sought to restrict the use of terms such as “steak,” “saucisse,” and “jambon” for products containing plant proteins, but both were suspended by the Conseil d'État before entering into force and were ultimately annulled on 28 January 2025, following the CJEU's ruling of 4 October 2024 (Case C‐438/23, *Protéines France and Others*), which held that such national restrictions conflict with the harmonized labeling framework of Regulation (EU) No 1169/2011. Globally, the United States takes a more flexible approach, while Canada and Australia have regulations like those of the EU, focusing on transparency. Current regulatory issues center on consumer protection, clear product labeling, and the authenticity of dairy products considering the growth of plant‐based alternatives, particularly for hybrid products combining animal and plant proteins (Shah et al. 2024).

## 9 Plant Protein Extraction Methods

Plant proteins occur as heterogeneous mixtures associated with various phytochemical compounds, including carbohydrates, oligosaccharides, lipids, and secondary metabolites. Therefore, extraction is usually required to obtain proteins with different profiles, qualities, and functionalities. Extraction methods can be divided into two categories: conventional methods (alkali, acid, and salt extraction and dry fractionation) and innovative methods (using ultrasound, pulsed electric fields, deep eutectic solvents, and enzyme‐assisted extraction). This section presents these two techniques to deepen the basic knowledge of extraction technologies (Tarahi 2024).

### 9.1 Conventional Extraction Methods and Their Limitations

Conventional methods, such as wet extraction (alkaline, acidic, saline) and dry fractionation, are most used to extract plant proteins (Table 5). In wet extraction, the proteins are extracted with aqueous or chemical solvents and then precipitated or recovered. Dry fractionation is based on the mechanical separation of proteins, starch, and other cellular components. After peeling and grinding, milling separates starch granules (≥ 20 μm) from protein fragments (1–3 μm) using techniques such as sieving, air separation, or electrostatic separation (Meenakshi Sundaram et al. 2024). Conventional methods have drawbacks such as toxic solvent residues, high energy consumption, environmental pollution, low yields, and changes in protein properties (digestibility, amino acid composition, bitter taste). For example, alkaline extraction can reduce digestibility, while acid extraction can alter solubility and gelling properties. During dry fractionation, proteins can remain in the starch fractions, which reduces the overall yield (Tang et al. 2024).

**TABLE 5:** Principles, conditions, protein content range, advantages, and disadvantages of conventional plant protein extraction methods.

Extraction method	Principle	Conditions	Protein content range	Advantages	Disadvantages/Limitations	References
Alkaline extraction	Solubilization of proteins at basic pH > pI (proteins become negatively charged)	Basic pH, followed by centrifugation	80%–94% (isolate, dry basis)	Most common method; simple, cost‐effective, and efficient	Reduces protein digestibility; can cause amino acid modification (e.g., lysinoalanine formation) and off‐flavor development at high pH	Sultan et al. (2024)
Acid extraction	Solubilization at acidic pH < pI, followed by precipitation through pH adjustment	Use of acidic solutions, pH control	80%–94% (isolate, dry basis)	Like the alkaline method; suitable for specific matrices	Alters protein solubility and gelling properties; can compromise functional performance	Sultan et al. (2024)
Saline extraction	Protein precipitation via the “salting‐out” effect (ions disrupt protein hydration)	Neutral salt solutions (NaCl, CaCl_2_, KCl), centrifugation	Variable; efficiency and protein content decrease at higher salt concentrations	Extracted proteins exhibit good solubility, foaming, emulsifying, and gelling properties	Yield and protein content decline at high salt concentrations; requires an additional desalting step before food‐grade use, adding cost	Day (2013), Akyüz et al. (2024)
Dry fractionation	Mechanical separation based on size and density of components after milling	Solvent‐free; uses sieving, air classification, electrostatic separation	40%–65% (concentrate, dry basis)	More sustainable process; solvent‐free; preserves native protein structure	Lower protein purity than wet extraction; residual protein remains trapped in starch fraction, reducing overall yield	Meenakshi Sundaram et al. (2024)

Beyond these processing drawbacks, wet and dry fractionation also yield fundamentally different product categories in terms of protein content, a distinction with direct consequences for dairy‐alternative formulation cost and application. Wet extraction typically produces isolates containing 80%–94% protein (dry basis), whereas dry fractionation generally produces concentrates in the 40%–65% range. Commercial legume protein ingredients are correspondingly categorized as flour (20%–25% protein), concentrate (50%–75%), or isolate (> 80%), depending on the fractionation route used (Schutyser et al. 2015). This gap is substantial even within a single legume species: faba bean isolate reaches 86.9% protein versus 63.9% for faba bean concentrate, and yellow pea isolate reaches 82.9% versus 50.2% for pea concentrate (Li et al. 2024). For dairy‐alternative applications, this creates a practical trade‐off distinct from the functional‐property trade‐offs: isolates are typically required where high protein content is a labeled nutritional claim (e.g., high‐protein plant‐based milk or yogurt), while concentrates, despite lower purity, are more cost‐effective and, as some studies report can retain a more native protein structure with comparable or superior gelling performance to isolates in certain matrices, making concentrates a viable choice where cost or structural functionality matters more than absolute protein content.

### 9.2 Innovative Methods for the Extraction of Plant Proteins

**FIGURE 4:**
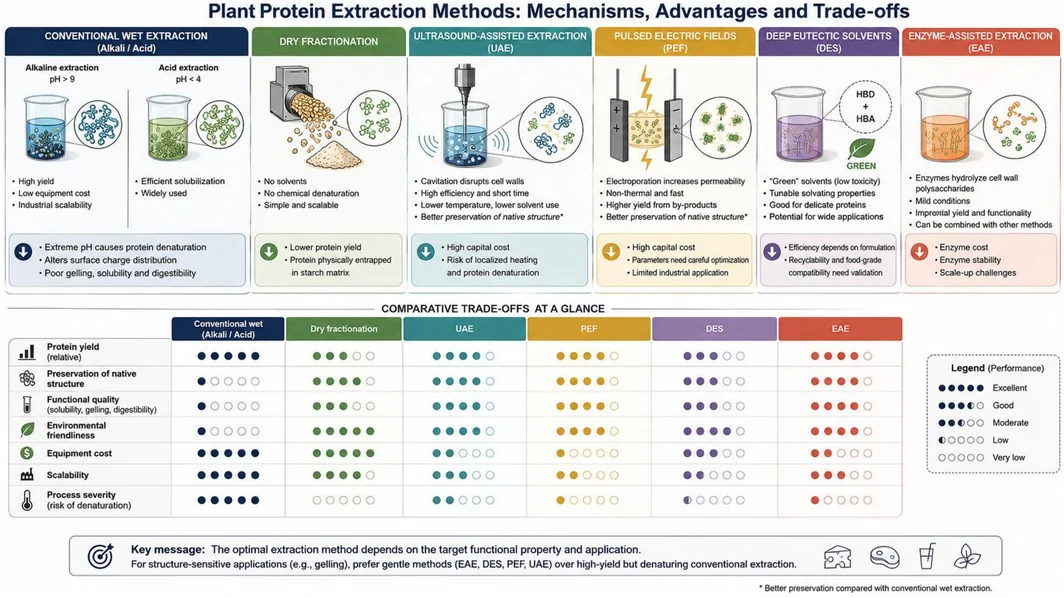
Comparative mechanisms, advantages, limitations, and performance trade‐offs of conventional and innovative plant protein extraction methods (alkaline/acid extraction, dry fractionation, ultrasound‐assisted extraction, pulsed electric fields, deep eutectic solvents, and enzyme‐assisted extraction), evaluated across protein yield, preservation of native structure, functional quality, environmental friendliness, equipment cost, scalability, and process severity.

The advancement of extraction technologies has led to the development of innovative methods that allow for improved extraction efficiency while respecting sustainability principles. New approaches include ultrasound‐assisted extraction (UAE), pulsed electric fields (PEF), deep eutectic solvents (DES), and enzyme‐assisted extraction (EAE). Each of these techniques offers specific advantages but also certain limitations that require adaptations (Tang et al. 2024). The extraction methods discussed above differ not only in mechanism but in the practical trade‐offs they impose between yield, structural preservation, cost, and scalability. Figure 4 summarizes these trade‐offs side by side, allowing the method best suited to a given target functional property or application to be identified immediately.

#### 9.2.1 Ultrasound‐Assisted Extraction (UAE)

UAE is based on applying high‐frequency ultrasound waves (generally between 20 and 100 kHz) that induce cavitation. This process causes microbubbles to form and implode, releasing mechanical and thermal energy that can rupture the matrix of the plant cells. This process facilitates the penetration of solvents into the cells and promotes the release of proteins. Compared to conventional methods, this technology is characterized by low energy consumption, high process speed, and lower treatment temperatures. However, unsuitable conditions can lead to oxidation or denaturation of the proteins, which limits their application on an industrial scale (Fatima et al. 2023).

#### 9.2.2 Extraction With the Aid of Pulsed Electric Fields (PEF)

In PEF, plant matrices are exposed to strong electrical impulses for very short periods. This treatment causes electroporation of cell membranes, increasing their permeability and promoting the release of intracellular macromolecules, including proteins. This non‐thermal approach is promising for the valorization of plant by‐products, as it significantly increases protein yield. However, the extraction parameters (field intensity, duration, pulse frequency, etc.) must be carefully optimized, and the application is not yet widespread in the field of plant proteins (Zhou et al. 2022).

#### 9.2.3 Use of Deep Eutectic Solvents (DES)

DESs are eutectic mixtures generally consisting of a hydrogen bond donor (HBD) and a hydrogen bond acceptor (HBA). These “green” solvents are valued for their non‐toxicity, biodegradability, chemical stability, and low cost. They can be used to extract a variety of bioactive compounds, including proteins, and replace conventional organic solvents. Their efficiency depends on several parameters, such as the HBD/HBA ratio, temperature, extraction time, and pH. Despite their potential, their recyclability, environmental impact, and compatibility with the food industry must be further investigated (Hernández‐Corroto et al. 2023).

#### 9.2.4 Enzyme‐Assisted Extraction (EAE)

EAE uses specific enzymes that can hydrolyze cell wall polysaccharides (cellulases, pectinases, amylases, etc.), facilitating the release of proteins into the solvent. This method is particularly suitable for heat‐sensitive compounds and improves the extracted proteins' yield and functional quality. It can be used alone or in synergy with other techniques. However, the cost of the enzymes and their stability are still obstacles to large‐scale application (Sharma, Sharma, et al. 2025).

Taken together, no single extraction method is universally superior; the optimal choice depends on which trade‐off a given application prioritizes. Conventional wet extraction remains dominant industrially because of its low equipment cost and scalability, but its reliance on pH extremes is precisely what compromises the functional properties discussed below: alkaline extraction denatures native protein conformation and reduces digestibility, while acid extraction alters surface charge distribution, explaining why proteins recovered by conventional routes often show weaker gelling and solubility profiles than those obtained by milder methods. Dry fractionation avoids solvent‐related denaturation entirely, but sacrifices yield, since a substantial protein fraction remains physically entrapped in the starch matrix rather than being chemically compromised: a fundamentally different limitation from that of wet extraction. Among the innovative methods, UAE and PEF both act through membrane disruption (cavitation vs. electroporation) rather than chemical denaturation, which is why they are generally reported to better preserve native protein structure and, by extension, functional performance; however, this advantage is offset by higher capital cost and, in the case of UAE, a narrower operating window before localized heating begins to denature the very proteins the method is meant to protect. DES and EAE occupy a different position on this trade‐off curve: both are comparatively gentle and environmentally favorable, but their extraction efficiency is highly formulation‐ or enzyme‐specific, making yields less predictable across different plant matrices than the more mechanically driven UAE/PEF approaches. This suggests that the extraction method should be selected not in isolation, but as a function of the downstream functional property the final application requires; for instance, gelling‐focused applications benefit more from structure‐preserving methods (EAE, DES, PEF) than from high‐yield but denaturing conventional extraction.

## 10 Functional Properties of Plant‐Based Proteins

### 10.1 Emulsifying Properties

The emulsifying properties of plant‐based proteins refer to their ability to stabilize oil–water mixtures by adsorbing at the oil–water interface, reducing the interfacial tension, and forming a protective layer around the oil droplets to prevent them from coalescing. This functionality is due to the amphiphilic nature of the proteins, which contain both hydrophilic (water‐attracting) and hydrophobic (oil‐attracting) regions, allowing them to act as natural emulsifiers in food and industrial applications (Lima et al. 2023).

Different legume protein isolates have various functional properties regarding protein content, emulsifying activity, and emulsifying stability. Mungbean protein isolates have protein contents of 81.5%–85.5%, with an emulsifying activity between 41.1% and 63.2% and a stability of 45.5%–62.8%. Pea protein isolates show significant differences, with a protein content of 83.6%–92.8%, an emulsifying activity of 4.6%–76.0%, and a stability between 11.0% and 78.1%. Soy protein isolates, both commercial and standard isolates, consistently exhibit high protein content (86.0%–92.4%) and strong emulsifying properties, with activity values up to 74.5% and stability of 81.2%. Chickpea protein isolates also show promising results, with protein contents between 63.9% and 89.1%, an emulsifying activity between 5.7% and 66.0%, and a stability of 10.9–82.9 min or percent (Sun et al. 2022). Lentil proteins, whether green, red, white, or yellow, generally have a high protein content (74.7%–91.2%), although emulsifiability (5.0%–72.0%) and stability (11.0%–67.0%) vary. Other legumes such as pigeon pea, cowpea, grass pea, and kidney bean also show remarkable functional properties. Pigeon pea and cowpea isolates have a protein content of over 86% and an emulsifying activity of over 69%, while the protein content of kidney beans is up to 94.7%, with moderate emulsifying activity (21.3%–23.7%) and stability (30.9–46.0 min) (Higa et al. 2024). Certain bean varieties, such as Akkus, Gembos, Simav, Hinis, and Bombay beans, for which no information on protein content is available, show emulsifying activities between 15.6 and 22.0 m^2^/g and stability values of up to 164.2 min. Overall, soy, pea, and chickpea proteins prove to be particularly effective emulsifiers that are suitable for use in food systems that require stable emulsions (Ma et al. 2022).

The wide variability reported above in pea protein emulsifying activity spanning 4.6%–76.0% versus soy's comparatively narrow and consistently high 74.5%–81.2% range is unlikely to reflect legume type alone. Soy protein's advantage is best explained by its higher proportion of the 7S (β‐conglycinin) globulin fraction, which unfolds more readily at the oil–water interface than the more compact 11S (glycinin) fraction that dominates several pea cultivars; this structural difference, rather than total protein content per se, is the more probable driver of interfacial performance, since pea isolates with comparable or higher protein purity than soy still underperform. The unusually broad ranges within a single legume (e.g., pea's 4.6%–76.0%) further point to extraction method as a major confounding variable rather than intrinsic species‐level variability: isolates recovered via harsher alkaline/isoelectric precipitation are more likely to show the lower end of this range, consistent with the denaturation effects discussed in the extraction section (Shanthakumar et al. 2022).

### 10.2 Foaming Properties

The foaming properties of plant‐based proteins relate to their ability to form and stabilize gas bubbles in liquid media, mainly by adsorption at air–water interfaces and the formation of interfacial films. These proteins reduce the surface tension and form viscoelastic layers that prevent the coalescence of the bubbles (Hu et al. 2025). Both steric hindrance and electrostatic repulsion between the protein‐coated bubbles achieve stabilization of the foam. Several factors influence the foam behavior. The protein structure plays a decisive role: hydrophobic amino acids and the hydrophobicity of the surface promote adsorption at the interface, while rigid structures, such as those with disulfide bonds, hinder flexibility and reduce foam formation. The pH value also plays a role. Lower electrostatic repulsion near the isoelectric point (pH 4–5) allows for denser protein packing, which increases foam stability (Zhang et al. 2022). Solubility is another key factor; while high solubility generally improves foaming, there are exceptions such as fava bean proteins, which have high foaming activity despite moderate solubility. Processing techniques such as homogenization can further improve foaming, especially for pea proteins, by increasing their solubility and surface activity (Cermeño et al. 2024). Among vegetable proteins, soy consistently shows superior foam stability and retains up to 98% of its foam due to its low‐fat content and optimal structural properties. Pea proteins show improved performance when homogenized or blended but are sensitive to destabilization by residual lipids. Fava bean proteins are characterized by their exceptional foaming properties, with expansion values reaching up to 2.278% due to their molecular flexibility (Sirison et al. 2021). Despite these promising properties, plant proteins often do not perform as well compared to animal proteins, primarily due to the presence of complex components such as polysaccharides and lipids and inherent structural rigidity. To overcome these challenges, strategies such as enzymatic hydrolysis or physical treatments such as ultrasound can be used to improve the flexibility of proteins. Mixing proteins with complementary interfacial properties, such as combining pea and soy proteins, is also promising. These improvements are particularly important for developing vegan desserts, whipped toppings, and other aerated foods (Lima et al. 2023).

The apparent contradiction noted above fava bean protein achieving high foaming activity despite only moderate solubility is informative rather than anomalous, and illustrates that foaming and solubility, while correlated, are governed by partially independent structural features. Foam formation depends primarily on how readily a protein unfolds and reorients at the air–water interface (a kinetic, structural‐flexibility property), whereas solubility reflects the thermodynamic balance of protein–water versus protein–protein interactions in bulk solution. A moderately soluble but structurally flexible protein, such as fava bean protein, can therefore still adsorb efficiently at the interface even if a smaller fraction of it is fully dissolved. This distinction also explains why soy's superior foam stability is attributed here to low fat content and structural optimality rather than solubility alone: residual lipids specifically compete with protein for interfacial space and destabilize the film regardless of how soluble the protein fraction is, which is consistent with pea protein's sensitivity to lipid‐driven destabilization despite otherwise favorable solubility.

### 10.3 Water and Oil Absorption Abilities

Plant‐based proteins have different water and oil holding capacities (WHC and OHC). These essential functional properties influence their performance in food formulations, especially meat and dairy alternatives (Ma et al. 2022). WHC refers to a protein's ability to absorb and bind water, while OHC indicates how well it can bind with oil. These properties affect the texture, juiciness, and overall mouthfeel of foods and are influenced by several factors, including protein source, purity, extraction and processing methods, and environmental conditions such as pH and temperature (Ma et al. 2022). For example, soy protein isolate, which is known for its high content of hydrophilic groups, can absorb up to 6.3 g of water per gram of protein, making it particularly effective at retaining moisture. Normally, WHC and OHC increase with higher protein purity and concentration. Proteins with greater surface hydrophobicity tend to have higher OHC, while proteins with more hydrophilic groups increase WHC (O'Flynn et al. 2021). Various techniques can improve these capacities, such as enzymatic treatment or high‐pressure processing. For example, in one study, sesame protein isolate treated under alkaline conditions with high pressure was found to have improved WHC and OHC. These properties are usually determined by mixing a certain amount of protein with distilled water or oil, followed by incubation and centrifugation (Gül et al. 2025). The retained liquid is then quantified and expressed as grams per gram of protein. However, the experimental conditions, such as protein concentration, mixing time, pH, temperature, and centrifugation speed, differ from study to study, making comparisons between different studies difficult. For example, the solubility and hydration of proteins vary with pH, with soy protein showing optimal water binding at a pH of 6 to 8 and a temperature of 40°C–70°C. In addition, centrifugation speeds of 1600–16,000 × *g* and centrifugation times of 10 to 30 min have been reported, which affect the final WHC values (Sajib et al. 2023).

Moreover, WHC can be determined for protein powders and after gel formation via heat or chemical cross‐linking. These capacities can also be measured directly in food matrices, such as plant‐based meats or cheeses. Legume protein isolates generally exhibit WHC values between 1.8 and 6.8 g/g and OHC between 3.5 and 6.8 g/g, with soy proteins often outperforming chickpea, pea, and lentil proteins due to structural differences (Kew et al. 2020).

The consistent outperformance of soy protein in WHC across studies is mechanistically linked to its higher density of hydrophilic surface groups relative to chickpea, pea, and lentil proteins, as noted above; but the magnitude of the reported ranges (1.8–6.8 g/g) is at least as attributable to methodological inconsistency as to genuine compositional differences. Because WHC/OHC assays are not standardized (centrifugation speeds alone range 40‐fold across cited studies, from 1600 to 16,000 × *g*), part of the apparent “source‐driven” variability documented here likely reflects protocol‐driven variability instead.

### 10.4 Gelling Properties

The gelling properties of plant proteins are crucial for developing semi‐solid plant‐based foods such as meat, cheese, and yogurt. When heated, these properties depend on the protein's ability to denature and transform into a three‐dimensional network that can retain water and resist flow. The least gelation concentration (LGC), defined as the minimum protein concentration required to form a stable gel, is commonly used as an indicator of gelling ability. Lower LGC values correspond to stronger gelling capacity. For example, proteins from *Lupinus albus* and cowpea with LGC values around 7%–7.5% have excellent gelling behavior, while proteins from *Lupinus angustifolius* and field bean have higher LGC values (20%–23%), indicating weaker gelling. These differences can be attributed to factors such as the thermostability of the proteins, the composition of the subunits (e.g., 7S vs. 11S globulins), the solubility, and the surface hydrophobicity (Shevkani 2023).

Ultrasound or thermal modification treatments can enhance hydrophobic interactions and improve gelation. Standard methods for evaluating LGC include heating protein solutions (2%–20%) to 100°C and observing gel formation during cooling and inversion. However, this method does not capture the gel texture, so additional techniques such as texture profile analysis and dynamic rheology are used to evaluate the strength, elasticity, and thermal stability of the gel under different conditions (Zhang, Ji, et al. 2025; Zhang, Herneke, et al. 2025).

The gap between *Lupinus albus* /cowpea (LGC ≈ 7%–7.5%) and *Lupinus angustifolius* /field bean (LGC ≈ 20%–23%) is a threefold difference in gelling efficiency between botanically related species, which underscores that gelling capacity is governed less by taxonomic origin than by the specific balance of 7S and 11S globulin subunits and their thermal stability. Proteins richer in the more thermolabile 11S fraction denature and cross‐link at lower concentrations, producing the lower LGC values (stronger gelling) seen in *Lupinus albus* and cowpea; species dominated by more thermostable subunit compositions require substantially higher protein concentrations to reach the same network‐forming threshold. This reinforces the point made in the extraction section: because gelation depends on the protein retaining sufficient native structural integrity to denature and reorganize in a controlled way upon heating, extraction methods that pre‐denature the protein (e.g., harsh alkaline extraction) would be expected to elevate LGC regardless of the source species' intrinsic gelling potential, a confound that the cited studies do not consistently control for.

### 10.5 Protein Solubility

Protein solubility is an essential technical‐functional property of plant‐based proteins strongly influenced by the balance between protein–water and protein–protein interactions, which are determined by parameters such as net surface charge, surface hydrophobicity, and structural conformation. Solubility reaches a minimum near the isoelectric point (pH 4–6), where electrostatic repulsion is minimal and protein aggregation is promoted by van der Waals, hydrophobic, and hydrogen bonding. In contrast, solubility typically increases at pH values far from the isoelectric point due to increased electrostatic repulsion; for example, values of over 80% at pH 8 have been reported for soy, chickpea, pea, and lentil proteins. The solubility profile also depends on the protein composition (e.g., vicilin‐rich fractions have a higher solubility than legumes), the distribution of hydrophilic and hydrophobic residues on the protein surface, and the processing method (Grossmann and McClements 2023). Techniques such as membrane filtration can enrich albumin content and improve solubility, while industrial‐scale drying methods can reduce solubility by altering protein structure and impairing rehydration. In addition, novel modification technologies, including ultrasound‐assisted extraction, high‐pressure homogenization, and enzymatic hydrolysis, have significantly improved solubility by reducing particle size and promoting structural unfolding. However, over‐treatment can lead to protein re‐aggregation and decreased solubility, highlighting the need for process optimization. It is also important to note that variations in experimental protocols such as dispersion conditions, incubation time and temperature, and protein quantification methods (e.g., Kjeldahl, Bradford, Lowry) can affect solubility results, making comparing different studies difficult. Despite these challenges, protein solubility remains a foundational property for predicting and enhancing functional attributes such as emulsification, foaming, and gelation, particularly in applications like plant‐based meat analogs and extenders, where a balance between soluble and insoluble protein fractions may be beneficial for achieving desirable textural characteristics (Suchintita Das et al. 2022).

The convergence of soy, chickpea, pea, and lentil proteins toward > 80% solubility at pH 8 despite their otherwise divergent emulsifying, foaming, and gelling profiles reported earlier in this section indicates that solubility is comparatively source‐insensitive once proteins are held far from their isoelectric point, whereas the more source‐specific functional differences emerge primarily near the isoelectric region and during interfacial or thermal processing. This has a practical implication for formulation: solubility alone is a poor predictor of which protein source will perform best in each application, since two proteins with near‐identical solubility profiles (e.g., soy and pea at pH 8) can diverge sharply in emulsifying or gelling behavior. As with the WHC/OHC data, the reliance on multiple non‐standardized quantification methods (Kjeldahl, Bradford, Lowry) noted here means that some of the residual variability attributed to “protein source” in the literature may in fact be a methodological artifact, a caveat that should temper direct cross‐study comparisons throughout this review.

### 10.6 Matching Protein Source and Extraction Method to Dairy‐Alternative Application

The preceding subsections establish that no single functional property reliably predicts a protein's overall suitability for dairy‐alternative formulation, and that extraction method is a recurring confounding variable across all five properties examined. Table 6 consolidates this evidence into a direct, application‐oriented framework, synthesizing the source‐, method‐, and application‐level findings developed throughout Sections 9 and 10 to indicate which protein source, processed by which method, is best suited to which dairy‐alternative category, and why. This framework is intended as a practical decision aid for formulators and a structured summary of the mechanistic reasoning presented above, rather than a substitute for source‐specific experimental validation.

**TABLE 6:** Recommended plant protein source and extraction method by dairy‐alternative application, based on functional performance evidence discussed in Sections 9 and 10.

Protein source	Dominant functional basis	Recommended extraction method	Best‐fit dairy application	Key limitation
Soy	High 7S content; consistent emulsifying (74.5%–81.2%) and foaming (up to 98% retention)	EAE or PEF (preserve native structure)	Creamers, beverages, whipped desserts	Allergen; conventional alkaline extraction erodes advantage
Pea	Highly variable emulsifying activity (4.6%–76.0%), extraction‐dependent	PEF or homogenization post‐extraction	Fortified beverages (non‐critical emulsification)	Requires controlled/verified extraction history; lipid‐sensitive foam
Fava bean	High foam expansion (up to 2278%) despite moderate solubility	UAE or EAE (preserve structural flexibility)	Aerated desserts, whipped toppings, soy‐free formats	Moderate solubility limits beverage‐clarity use
Chickpea	Moderate‐to‐good emulsifying (5.7%–66.0%); good WHC contribution	EAE (documented efficiency for legume matrices)	Yogurt bases (blended), beverages	Wide activity range increases QC burden
*Lupinus albus* /cowpea	Low LGC (7%–7.5%); strong gel network at low protein concentration	Gentle methods only (DES, EAE) — harsh extraction elevates LGC	Cheese and yogurt analogs (structural set)	Thermal sensitivity risks syneresis if over‐processed
*Lupinus angustifolius* /field bean	High LGC (20%–23%); weaker gelling	Any (gelling not primary use case)	Beverage/protein‐fortification role rather than structured products	Requires higher protein input for equivalent gel firmness

Table 6. Here's Table 6 as a rendered markdown table—this will copy cleanly as an actual table into Word, Google Docs, or Excel in most browsers/apps (select and copy directly from the rendered table below, not the raw text).

### 10.7 Sensory Properties

Beyond the instrumental functional properties discussed above, sensory acceptability is a decisive and often limiting factor in plant‐protein dairy‐alternative formulation. Most legume and pulse proteins exhibit characteristic “beany”, “grassy”, and bitter off‐notes generated primarily through the lipoxygenase hydroperoxide lyase pathway, in which oxidation of polyunsaturated fatty acids produces volatile aldehydes (notably hexanal) alongside non‐volatile contributors such as saponins (astringency) and isoflavones/phenolics (bitterness) (Xiang et al. 2023). Because these compounds originate in the raw material and are further shaped by extraction and processing conditions, sensory quality is mechanistically linked to the same variable's extraction method, thermal treatment, and protein source discussed throughout Sections 9 and 10 rather than being an independent formulation concern.

This is well documented directly in dairy‐alternative matrices. In oat‐based milk alternatives, fortification with pea protein isolate produced a significantly bitter and metallic taste, an astringent aftertaste, and increased overall aroma intensity linked to elevated volatile compound levels, while potato protein fortification caused fewer flavor changes but increased powdery mouthfeel, mouthcoating, and a detectable color change both proteins also produced a “staler” flavor profile after UHT processing specifically (McCarron et al. 2024). This indicates that sensory impact is protein‐source‐specific in ways that do not necessarily correspond to the functional properties discussed earlier: potato protein, largely absent from the functional‐property discussion above, introduces distinct sensory liabilities (mouthcoating, particle‐size‐driven texture change) that a purely functional evaluation would entirely miss.

Processing offers a partial mitigation route. In pea protein yogurt, fermentation with lactic acid bacteria significantly reduced hexanal and other pea‐odor volatiles likely through lipoxygenase inactivation during heat treatment combined with aldehyde‐metabolizing enzyme activity during fermentation while simultaneously generating desirable fermented‐food aroma compounds, meaningfully improving the flavor profile relative to unfermented pea protein dispersions (Xiang et al. 2023; Zhang, Ji, et al. 2025). This suggests that fermented dairy‐analog formats (yogurt, kefir‐style products) may have a structural sensory advantage over unfermented formats (beverages, creamers) that current formulation strategies do not fully exploit and reinforces a broader point from Section 9: processing choices in plant‐protein dairy alternatives serve simultaneous digestibility, functional, and now sensory‐mitigation roles, rather than being independent process parameters selected for a single objective.

## 11 Conclusion

This review demonstrates that no single plant protein source or extraction method is universally optimal for dairy‐alternative formulation; rather, protein selection must be matched to the specific functional demand of the target application. Structural distinctions particularly the relative proportion of 7S versus 11S globulin fractions consistently explain why soy and pea proteins diverge in emulsifying and gelling performance despite comparable protein purity, while extraction method emerges as a recurring confounding variable across the functional‐properties literature, since harsher alkaline or acid extraction routes measurably compromise the same structural integrity that determines gelation and interfacial behavior. Protein‐quality comparisons using DIAAS confirm that only soy protein isolate approaches “good” status among plant sources, with pea, oat, wheat, and rice proteins each limited primarily by lysine or sulfur amino acid content reinforcing that blended, rather than single‐source, formulations are generally required to approach dairy‐protein amino acid completeness. At the same time, this review's cross‐study comparisons reveal a persistent limitation in the underlying evidence base itself: the absence of standardized assay protocols (e.g., centrifugation speed, protein quantification method) across functional‐property studies means that some proportion of the reported “source‐driven” variability likely reflects methodological inconsistency rather than genuine compositional differences, a caveat that should temper direct comparisons until standardized testing protocols are more widely adopted. Collectively, these findings position plant protein selection for dairy alternatives as a multi‐criteria decision balancing protein quality, extraction‐preserved functionality, and dairy‐specific performance requirements rather than a search for a single “best” plant protein source.

## 12 Directions for Future Research

Building on the comparative gaps identified throughout this review, four research priorities emerge as most likely to advance the field. First, the absence of standardized functional‐property assay protocols identified in this review (Sections 9 and 10) represents a foundational obstacle: inter‐laboratory validation studies establishing reference protocols for emulsification, gelation, and water/oil‐holding capacity are needed before source‐ and method‐dependent comparisons across the existing literature can be considered fully reliable. Second, given the structural basis for functional divergence highlighted here particularly the 7S:11S globulin ratio targeted structure–function studies that deliberately vary this ratio (through selective fractionation or blending) would test whether functional performance can be predictably engineered rather than only observed post hoc. Third, since DIAAS comparisons in this review show that most single plant protein sources fall below the FAO “good” quality threshold, pilot‐scale studies validating specific blending ratios (e.g., cereal‐legume pairings) for amino acid complementarity in real dairy‐analog matrices, rather than in isolated protein systems, are needed to confirm that laboratory‐level complementarity translates to finished‐product nutritional equivalence. Fourth, industrial‐scale techno‐economic and life‐cycle assessments of the emerging extraction technologies discussed here (ultrasound‐, pulsed‐electric‐field‐, and enzyme‐assisted extraction) remain scarce relative to bench‐scale functional data, and are necessary to determine whether their demonstrated structural advantages persist, and remain cost‐competitive, at production volumes. Progress on these fronts will depend on continued interdisciplinary collaboration among food chemists, process engineers, and regulatory bodies, alongside consumer research to guide the sensory and labeling adaptations needed for wider market acceptance of plant‐based dairy alternatives.

## Author Contributions

**Boutheina Ben Akacha:** conceptualization, methodology, validation, formal analysis, investigation, data curation, writing – original draft, writing – review and editing. **Monika Michalak:** conceptualization, data curation, validation, formal analysis, investigation, writing – review and editing, visualization. **Miroslava Kačániová:** formal analysis, validation, data curation, writing – review and editing. **Maciej Ireneusz Kluz:** methodology, validation, formal analysis, writing – review and editing. **Wirginia Kukula‐Koch:** validation, investigation, writing – review and editing. **Wojciech Koch:** validation, investigation, writing – review and editing. **Ramona Suharoschi:** methodology, formal analysis, writing – review and editing. **Rania Ben Saad:** validation, formal analysis, data curation, writing – review and editing. **Stefania Garzoli:** validation, formal analysis, writing – review and editing, supervision. **Anis Ben Hsouna:** conceptualization, methodology, validation, formal analysis, data curation, visualization, writing – review and editing, supervision, project administration.

## Funding

This work was funded by grants provided by the Tunisian Ministry of Higher Education and Scientific Research (Program contract 2022–2026).

## Conflicts of Interest

The authors declare no conflicts of interest.

## Data Availability

Data will be made available on request.
